# Telomere Length Variation in Model Bryophytes

**DOI:** 10.3390/plants13030387

**Published:** 2024-01-28

**Authors:** Liia R. Valeeva, Anastasia V. Sannikova, Nadiya R. Shafigullina, Liliia R. Abdulkina, Margarita R. Sharipova, Eugene V. Shakirov

**Affiliations:** 1 Institute of Fundamental Medicine and Biology, Kazan Federal University, Kazan 420008, Republic of Tatarstan, Russia; anastasya.sannikova@bk.ru (A.V.S.); nigmatullinalili@mail.ru (L.R.A.);; 2 Department of Biological Sciences, College of Science, Marshall University, Huntington, WV 25701, USA; 3 Institute of Environmental Sciences, Department of General Ecology, Kazan Federal University, Kazan 420008, Republic of Tatarstan, Russia; 4 Department of Biomedical Sciences, Joan C. Edwards School of Medicine, Marshall University, Huntington, WV 25755, USA

**Keywords:** TRF analysis, ITS, *Physcomitrium*, *Ceratodon*, *Marchantia*, *Sphagnum*, dioecious

## Abstract

The ends of linear chromosomes of most eukaryotes consist of protein-bound DNA arrays called telomeres, which play essential roles in protecting genome integrity. Despite general evolutionary conservation in function, telomeric DNA is known to drastically vary in length and sequence between different eukaryotic lineages. Bryophytes are a group of early diverging land plants that include mosses, liverworts, and hornworts. This group of ancient land plants recently emerged as a new model for important discoveries in genomics and evolutionary biology, as well as for understanding plant adaptations to a terrestrial lifestyle. We measured telomere length in different ecotypes of model bryophyte species, including *Physcomitrium patens*, *Marchantia polymorpha*, *Ceratodon purpureus*, and in *Sphagnum* isolates. Our data indicate that all analyzed moss and liverwort genotypes have relatively short telomeres. Furthermore, all analyzed ecotypes and isolates of model mosses and liverworts display evidence of substantial natural variation in telomere length. Interestingly, telomere length also differs between male and female strains of the dioecious liverwort *M. polymorpha* and dioecious moss *C. purpureus*. Given that bryophytes are extraordinarily well adapted to different ecological niches from polar to tropical environments, our data will contribute to understanding the impact of natural telomere length variation on evolutionary adaptations in this ancient land plant lineage.

## 1. Introduction

Telomeres are evolutionarily conserved structures found in most eukaryotic genomes at the ends of linear chromosomes. Telomeres play a major role in cellular homeostasis by providing protection against chromosome aberrations and contributing to organismal life span control. Telomeric DNA is conserved across eukaryotic evolution with telomere repeats in most species having a variation of GT-rich sequences [1]. Although the important roles of telomeres were originally highlighted by classical studies in plants [2], the biology of plant telomeres is still largely understudied. Historically, the main model system to study plant telomere biology was the small flowering plant *Arabidopsis thaliana* [3,4,5]. Detailed characterization of Arabidopsis telomere biology by many research groups over the past decades has uncovered intriguing contributions of telomeres and telomerase to plant genome stability, genomic consequences of telomere failure, and the molecular basis of the extraordinary tolerance of plants to telomere dysfunction [6]. However, some of the current research efforts are beginning to shift towards new model systems that promise a fresh look at understanding plant telomere biology through the lenses of evolution. These studies are also assisted by the rapid development of new technologies, such as the single-cell and nanopore sequencing strategies [7,8], which open new doors to the analysis of plant telomeres in a broader set of plant evolutionary lineages.

Bryophytes represent a group of non-vascular plants that diverged relatively early from other land plant lineages [9,10]. Despite their apparent simplicity (i.e., relatively few cell types), they evolved distinct developmental adaptations that make them uniquely suited for studies of divergent and convergent evolutionary features. The bryophytes contain three major divisions: mosses (Bryophyta), liverworts (Marchantiophyta), and hornworts (Anthocerotophyta) [11,12]. The moss *Physcomitrium patens* (known as *Physcomitrella patens* prior to 2019) was among the “second-generation” plants adopted as model organisms [13]. *P. patens* was also the first non-vascular plant with a sequenced genome, making it one of the best plant model systems to study evolutionary genetics, plant development, and adaptations to life on land [14]. Several other bryophytes have also been studied, including the model liverwort *Marchantia polymorpha* [13]. The *M. polymorpha* genome is characterized by low genetic redundancy due to the lack of recent genome duplications. Coupled with its ease of propagation in laboratory conditions, these advantages make *M. polymorpha* an ideal system for functional gene analysis [15,16,17]. Other more recently developed model bryophyte systems suitable for genomic and evolutionary studies include *Ceratodon purpureus* and *Sphagnum* species, as well as model hornworts *Anthoceros agrestis* and *A. punctatus* [18,19,20].

Bryophytes also represent a promising evolutionary lineage to study plant telomere biology. First, the predominant phase of lifecycle for all bryophytes is haploid, easily distinguishing them from all other land plants [21]. In comparison to diploid plant models, the haploid genome allows for quick and straightforward transgenic manipulations. For instance, mutant *P. patens* plants can be easily generated within months using highly efficient homologous recombination mechanisms [22,23]. Second, unlike most flowering plants, many bryophyte species are dioecious [24], providing a promising route for the identification of putative telomere biology genes associated with sex chromosomes. Finally, given that bryophytes likely diverged from other land plant lineages around 500 million years ago [25], they are thought to have evolved a number of distinct adaptations to the terrestrial lifestyle, including unique approaches to protect chromosomal DNA from environmental damage [26].

Unlike the situation with the flowering plants [27,28], very few bryophyte species have previously been investigated for specific features of telomere biology. All bryophytes analyzed so far harbor the canonical Arabidopsis-type telomere repeats TTTAGGG [29,30,31,32], and in a few species, the composition of telomeric repeats can be deciphered from the whole-genome sequencing data [18,20,33,34]. The moss *P. patens* was previously established as a new model for telomere biology and a counterpoint to Arabidopsis by investigating the evolutionary conservation and functional roles of the telomere binding protein POT1 [30]. Deletion of the *P. patens POT1* gene resulted in severe developmental defects, sterility, and substantial telomere shortening with extended G-overhangs followed by end-to-end chromosome fusions. Telomere dynamics and telomerase activity were also extensively evaluated in *P. patens* [31], and the utility of this moss system for characterizing telomere biology in the context of multiple mutations in DNA damage and repair pathways was established [35]. Furthermore, the functional roles of the recently identified telomerase RNA (TR) gene [36,37] in telomerase activity and telomere maintenance were also recently established in *P. patens* [38]. However, so far, no functional molecular or reverse genetics studies on telomere biology genes have been conducted in any other model bryophyte species.

Here, we evaluate telomere length diversity and sequence in several previously uncharacterized bryophytes species, including *Ceratodon purpureus* and *Sphagnum* isolates. Using terminal restriction fragment assays, we detect substantial natural variation in telomere length between different ecotypes of all bryophyte species investigated, including the previously characterized model moss *P. patens*. We also, for the first time, analyze telomere length in male and female strains of two dioecious bryophyte species, the model liverwort *M. polymorpha* and moss *C. purpureus*. Coupled with the high levels of genetic variation in natural accessions of model bryophytes, our results pave the way for the future establishment of this early diverging division as a powerful avenue for characterizing genetic architecture of telomere length control in land plants.

## 2. Results

### 2.1. Telomere Length Varies in Physcomitrium patens Ecotypes

**Figure 1:**
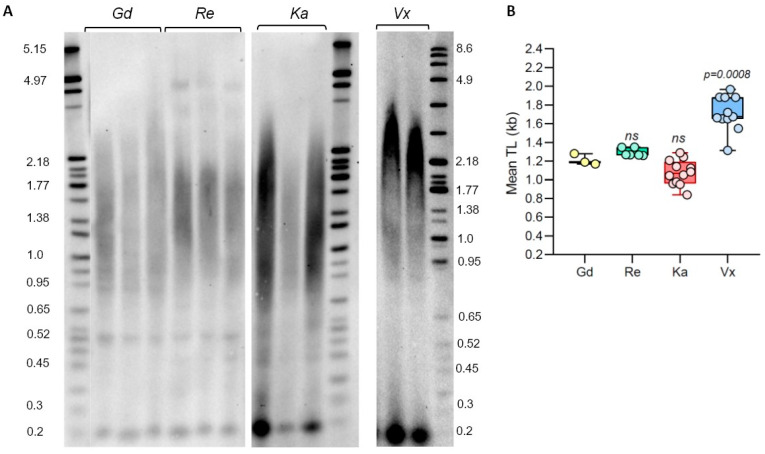
Telomere length variation in *P. patens* ecotypes. (**A**) TRF Southern blot for *P. patens* Gd, Re, Ka, and Vx ecotypes. Molecular weight DNA markers (in kb) are shown. (**B**) Telomere length (mean TRF) distributions in ≥3 biological replicates of each genotype are shown in boxplots. Data points represent mean TRF values from individual moss plates (biological repeats) analyzed with WALTER. Whiskers indicate maximum to minimum values; boxes represent the lower and upper quartiles (25 and 75%); horizontal lines represent medians of the mean TRF values. Significance *p*-value vs. Gd is shown; ns—no significant differences.

Although many different *P. patens* accessions have been described in the literature, the first sequenced Gransden ecotype (Gd, originally isolated in UK) is still the accession of choice for many molecular genetics and physiological experiments [26,33]. However, several other genetically diverse accessions are being increasingly used in *P. patens* research, which often provide important biological advantages, such as the production of more sporophytes and better applicability to multi-generational studies [39]. In the earlier moss studies, telomere length (TL) was only measured in two *P. patens* ecotypes, Gd [30,31] and Villersexel (Vx, France) [30]. To extend TL analysis to additional *P. patens* accessions, we performed TRF on Gd, Vx, and two other ecotypes with confirmed genotype differences [14,40]: Reute (Re, Germany) and Kaskaskia (Ka, USA) (Figure 1). Analysis of the Gd accession verified earlier data that TL in this ecotype is relatively short, with mean TRF being 1.21 ± 0.06 kb (Supplementary Table S1). Telomere length in the Re genotype DNA digested with TruI1 was not significantly different from the Gd telomeres, with mean TRF in this accession being 1.29 ± 0.04 kb. Telomeres in the Ka ecotype are slightly shorter than in Gd, with the mean TRF value of 1.08 ± 0.13 kb (Figure 1, Supplementary Table S1). The longest telomeres were detected in the Vx ecotype: mean TRF in this accession is 1.71 ± 0.19 kb, confirming results from our earlier study [30]. Taken together, our data indicate that telomere length varies between analyzed *P. patens* ecotypes, with Kaskaskia telomeres being the shortest and Vx telomeres being the longest. However, this TL variation is not as substantial as in natural accessions of the model flowering plant *A. thaliana* [41].

**Figure 2:**
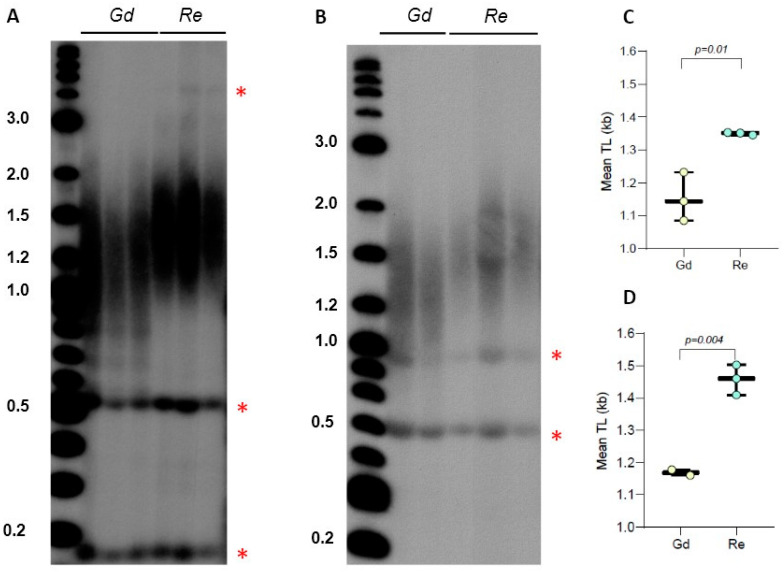
Telomere length in *P. patens* Reute and Gransden ecotypes. (**A**) TRF Southern blot for Gd and Re ecotype DNA digested with TruI1 and RsaI enzymes. Molecular weight DNA markers (in kb) are shown. Asterisks indicate positions of interstitial telomeric DNA bands. (**B**) TRF Southern blot for Gd and Re DNA digested with HaeIII, MboI, and AluI enzyme combinations. (**C**,**D**) Mean TRF distributions in biological replicates of each genotype are shown in boxplots for digestion with TruI1 and RsaI (**C**) and HaeIII, MboI, and AluI (**D**) enzymes. Data points represent mean TRF values from individual moss plates (biological repeats) analyzed with WALTER. Significance *p*-value vs. Gd is shown.

We previously detected the presence of a sharp 0.5 kb band in *P. patens* TRF gels for Gd accession and showed that this DNA represents a type of interstitial telomeric sequence (ITS), which was insensitive to BAL31 exonuclease digestion, unlike the true telomeric signal from chromosomal ends [30]. In Figure 1, we also detected the presence of similar size bands in lanes containing TruI1-digested DNA from Re, though lanes with Vx and Ka DNA did not show this signal. To test if the pattern of this ITS band migration will change when genomic DNA is digested differently, we examined TRF profiles of Gd and Re DNA samples digested with the combination of TruI1 and RsaI restriction enzymes. Interestingly, the position of this ITS band in the gel did not change, suggesting that this cross-hybridizing region of genomic DNA does not contain RsaI sites (Figure 2A). Furthermore, this band remained intact even when Gd and Re DNA samples were treated with the combination of HaeIII, MboI, and AluI enzymes (Figure 2B). In addition, in Re DNA samples treated with TruI1 only or with the combination of RsaI and TruI1, we noticed the appearance of a weak band at 4 kb (Figure 1A and Figure 2A), while in HaeIII-, MboI-, and AluI-treated Re samples, another sharp band at ~0.9 kb was detectable (Figure 2B). Moreover, in all four DNA samples treated with TruI1, a sharp ITS band was detected at ~0.2 kb (Figure 1A), which disappeared in HaeIII-, MboI-, and AluI-treated, but not in RsaI- and TruI1-treated Gd and Re samples (Figure 2A,B). These observations suggest a complex nature of ITS sequences in *P. patens* ecotypes. Overall, we note that the combination of two or three restriction enzymes in TRF analysis appears to better separate the true telomeric signal in the Reute ecotype, making the difference in TL between Gd and Reute ecotypes more apparent and statistically significant (Figure 2C,D, Supplementary Table S2). Thus, we suggest utilizing multiple enzyme digestion when comparing telomeric signals in different *P. patens* genotypes.

### 2.2. Telomere Length Analysis in Dioecious Bryophyte Species

*Ceratodon purpureus* is a model moss species that rapidly gains popularity in plant development and evolution studies. Unlike *P. patens*, which is monoecious, *C. purpureus* is dioecious, with genomes and transcriptomes of the two reference strains GG1 (female) and R40 (male) recently characterized [18,42]. Though telomere length in *C. purpureus* strains has not been explored before, molecular characterization of *C. purpureus* telomeres can open new directions in the analysis of sex-associated differences in plant telomere biology.

Evaluation of WGS data through the TeloBase database [27] indicated that *C. purpureus* harbors the canonical plant-like telomere repeat, TTTAGGG. We analyzed TL in four natural isolates of *C. purpureus* collected from three different geographical locations: R40 (male strain, New York, USA), GG1 (female strain, Austria), B150 and B190 (female and male lines, respectively, Alaska, USA). TRF analysis using TruI1 endonuclease revealed a highly heterogeneous profile of telomeric signal, with multiple distinct bands and broadly distributed smears (Supplementary Figure S1A). Mean TRF values in *C. purpureus* accessions varied from the shortest 0.68 ± 0.04 kb in the GG1 ecotype to the longest 1.15 ± 0.14 kb in the B190 accession (Supplementary Table S3). Interestingly, we did not find sex-specific correlations in mean TRF values between female and male lines: the female Alaskan isolate B150 had longer telomeres (*p* = 0.03) than the male Alaskan isolate B190, as well as longer than the other male line R40 (*p* = 0.002) (Supplementary Figure S1B). On the other hand, mean TRF in the second female GG1 line was shorter than in any other line, indicating that TL in the two analyzed female strains is located on the opposite ends of the telomere length spectrum specific for this species.

**Figure 3:**
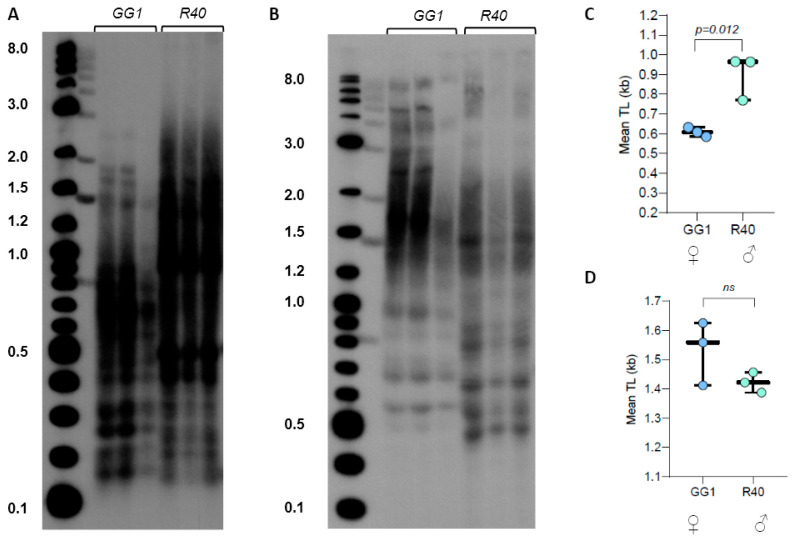
Telomere length in *C. purpureus* GG1 (female) and R40 (male) ecotypes. (**A**) TRF Southern blot for DNA digested with TruI1 and RsaI enzymes. Molecular weight DNA markers (in kb) are shown. (**B**) TRF Southern blot for DNA digested with HaeIII, MboI, and AluI enzyme combinations. (**C**,**D**) Mean TRF distributions in ≥3 biological replicates of each genotype are shown in boxplots for digestion with TruI1 and RsaI (**C**) and HaeIII, MboI, and AluI (**D**) enzymes. Significance *p*-value is shown; ns—no significant differences.

Given the very broad distribution pattern of TRF signals in *C. purpureus* DNA digested with TruI1, we next examined TRF profiles of GG1 and R40 (the two strains with sequenced genomes) DNA samples digested with the combination of TruI1 and RsaI restriction enzymes. The double digestion resulted in better separation of TRF signals (less heterogeneous) with R40 telomeric signals clearly appearing longer than the telomeric DNA in GG1 (Figure 3A,C). However, when DNA samples were treated with the combination of three enzymes (HaeIII, MboI, and AluI), the mean TRF value for GG1 was slightly longer than the value for R40 (Figure 3B,D, Supplementary Table S4). We note that the triple digest of telomeric fragments in *C. purpureus* ecotypes may be more preferred for the future analysis of telomere length in this species, as this combination of enzymes produces a longer TRF size range that may be technically easier to quantify with both TeloTool and WALTER telomere length analysis tools [43].

**Figure 4:**
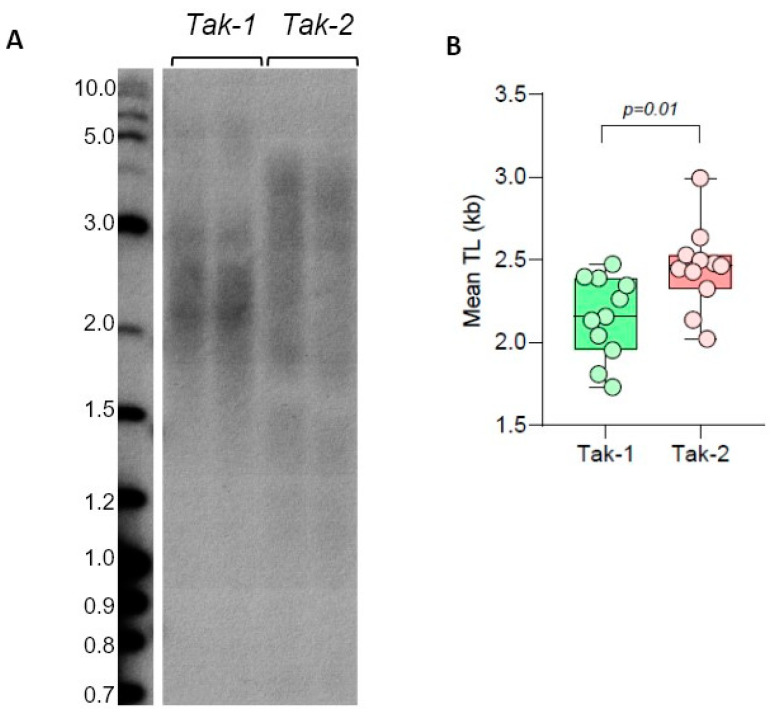
Telomere length in *M. polymorpha* ecotypes. (**A**) TRF Southern blot for Tak-1 (male) and Tak-2 (female) lines. Molecular weight DNA markers (in kb) are shown. (**B**) Mean TRF distributions in biological replicates of each genotype are shown in boxplots. Significance *p*-value is shown.

We next analyzed TL distribution in male and female strains of another dioecious bryophyte, the model liverwort *Marchantia polymorpha*. We have previously shown that telomeric DNA in the *M. polymorpha* Tak-1 strain also consists of Arabidopsis-like TTTAGGG repeats with mean TRF being ~2 kb. We next compared TL in Tak-2 (female strain) and Tak-1 (male strain), which are the reference genotypes with recently analyzed genomes [32,44]. TRF analysis of *M. polymorpha* telomeres indicated that mean TRF values in Tak-1 (2.15 ± 0.25 kb) and Tak-2 (2.45 ± 0.25 kb) lines were different (Figure 4, Supplementary Table S5), with the female strain having longer telomeres. Future validation of sex-specific differences in *M. polymorpha* strains will require analysis of additional isolates of this liverwort. Overall, we conclude that model dioecious bryophyte species, *C. purpureus* and *M. polymorpha*, show substantial variation in telomere length between various accessions, though the observed differences in TL do not currently support sex-specific correlations among analyzed genotypes. Additionally, we note that telomeres in the liverwort *M. polymorpha* are longer than in all analyzed ecotypes of the model mosses *P. patens* and *C. purpureus*.

### 2.3. Sphagnum Telomeres Contain Canonical Plant Telomeric Sequence TTTAGGG

The *Sphagnum* (peatmoss) genus belongs to the *Sphagnopsida* class that likely diverged from other bryophytes 250–350 mya [45]. *Sphagnum* species are found throughout the world and are quickly becoming powerful model organisms for plant ecological and evolutionary genomics studies [46]. However, telomere length in *Sphagnum* species has not previously been evaluated. To assess the level of TL variability in *Sphagnum*, we analyzed *Sphagnum fallax* MV (an established laboratory strain) and two natural isolates, *Sphagnum girgensohnii* and *Sphagnum* sp., collected in Ekaterinburg and the Republic of Mari El, Russia, respectively.

**Figure 5:**
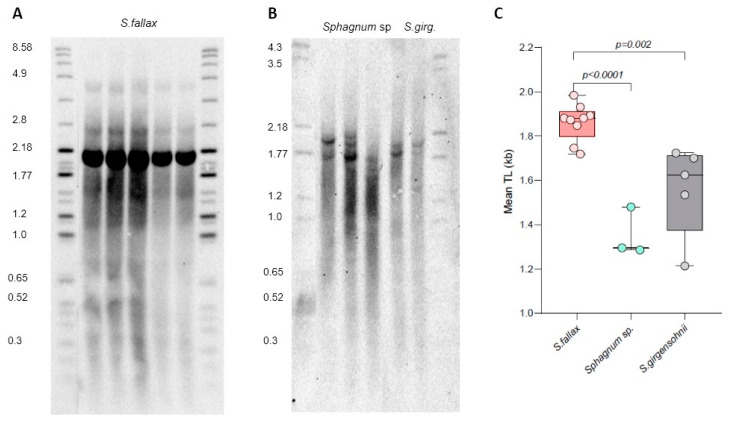
Telomere length variation in *Sphagnum* isolates. TRF Southern blots for *S. fallax* MW ecotype (**A**), and *Sphagnum* sp. and *Sphagnum girgensohnii* (*S. girg*) isolates (**B**). Molecular weight DNA markers (in kb) are shown. (**C**) Telomere length (mean TRF) distributions in ≥3 biological replicates of each genotype are shown in boxplots. Significance *p*-values vs. *S. fallax* are shown.

TRF analysis revealed variable telomere length in the three *Sphagnum* isolates (Figure 5). Mean TRF differed in *S. fallax* (1.86 ± 0.08 kb), *S. girgensohnii* (1.56 ± 0.21 kb), and *Sphagnum* sp. (1.35 ± 0.11 kb) (Supplementary Table S6), implying natural variation in telomere length between different *Sphagnum* species. In addition to the typical telomeric smear, in *S. fallax* we also detected a very strong band of high intensity at ~2.1 kb (Figure 5A), which was not nearly as strong in the other two isolates. To evaluate if this band also corresponds to chromosome ends and not to interstitial telomeric sequences, DNA was preincubated (prior to digestion by Tru1I) with BAL31 non-specific exonuclease that preferentially degrades DNA ends versus more internal genomic regions (Supplemental Figure S2). With continued BAL31 incubation, telomeric signals became weaker, and by 120 min, the smear disappeared almost completely. Signal intensity of the ~2.1 kb also decreased after 60 min of incubation, suggesting its sensitivity to BAL31 treatment, though its telomeric nature cannot yet be established unambiguously. Overall, the BAL31 data further confirmed that the terminal telomeric DNA of *S. fallax* is composed of TTTAGGG repeats. Thus, we conclude that similar to all other analyzed bryophytes ([29,30,31] and this study), members of the *Sphagnum* genus are also characterized by the canonical plant telomeric sequence TTTAGGG.

### 2.4. Telomere Length Stability in Long-Term Moss Cultures

Most of the moss lifecycle is spent in the haploid form, starting with when the actively dividing protonemata develops rapidly from the spore to allow plant growth over longer distances, followed by the later development of the more mature gametophore tissue [14]. In standard laboratory culture conditions, both *P. patens* and *C. purpureus* are maintained and propagated as protonema or gametophores for very long periods of time, often for months with regular weekly passages, which over time could lead to accumulation of somatic mutations [40]. In flowering plants, telomere length does not appear to change over time in different cells, tissues, and organs during plant development [4,47]. Similarly, TL in *P. patens* protonema (Gd ecotype) was shown to not change during the first seven days after passaging [31]. However, longer growth periods for *P. patens* genotypes and other moss species have not been previously analyzed for telomere length dynamics in protonema or gametophore tissues.

**Figure 6:**
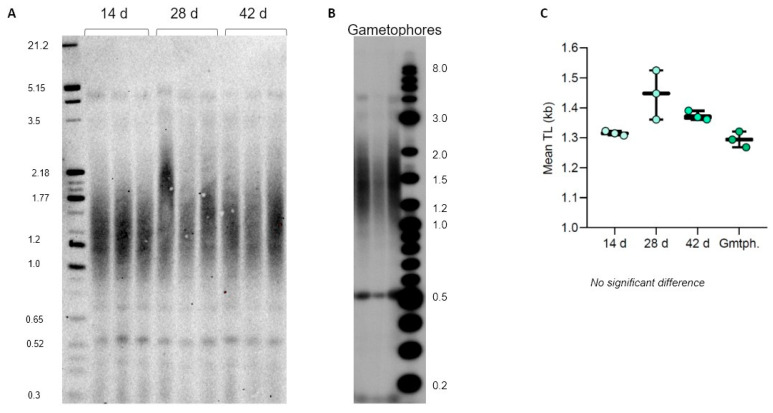
Telomere length dynamics in *P. patens* Reute ecotype. TRF Southern blots for 14-, 28-, and 42-day protonema cultures (**A**) and 2-month-old gametophores (**B**). (**C**) Telomere length (mean TRF) distributions in 3 biological replicates of each culture are shown in boxplots. No significant changes in telomere length are detected.

We first evaluated TL dynamics of *P. patens* protonema (Reute ecotype) grown on plates for 14, 28, and 42 days. Interestingly, we observed no mean TRF changes in vegetatively grown protonema tissue for the entirety of the cultivation (Figure 6A,C), suggesting no telomere length change due to multiple cell divisions associated with the rapid growth of protonema. Furthermore, the same TL was observed in 2-month-old gametophores propagated on plates (Figure 6B,C), suggesting no TL changes associated with transition to a more developmentally advanced tissue. We next followed TL dynamics in *C. purpureus* GG1 and R40 protonema tissues grown on plates for 14, 28, and 42 days. Similar to the situation observed for *P. patens*, both GG1 and R40 *C. purpureus* lines maintained telomere length at the same level during the entire protonema cultivation period (Supplementary Figure S3). Taken together with the previously published data on flowering plants, we conclude that telomere length remains relatively stable over time in different tissues of vegetatively growing plants—a conserved feature of telomere biology throughout major lineages of land plant evolution.

## 3. Discussion

### 3.1. Intra-Species Variability in Telomere Length in Model Bryophytes 

The length of telomeric tracts is one of the main functional characteristics of telomere biology in all organisms. In plants, telomere length can vary several fold: from relatively short telomeres of 0.3 kb in the unicellular green algae *Chlamydomonas reinhardtii* [48] to up to 150 kb in tobacco (*Nicotiana tabacum*) and up to 80 kb in barley (*Hordeum vulgare*) [49,50]. While much effort in the past was focused on understanding telomere length homeostasis in the flowering plants and, specifically, in the model plant *A. thaliana*, less attention was given to evaluating telomere length in the early diverging land plant lineages. Although current technological advances allow for powerful telomere length estimation algorithms using whole-genome sequencing efforts [51], experimental confirmation of in silico data is often necessary to validate and support computational approaches [41].

Among the few previously evaluated clades of the non-seed vascular plants (ferns and fern allies), telomere length was experimentally examined in the lycophyte *Selaginella moellendorffii* [52] and fern *Psilotum nudum* [29]. For the non-vascular plants, telomere length was previously analyzed in several mosses and liverworts, including the model moss *P. patens* [30,31], moss *Barbula unguiculata* [29], liverworts *Marchantia paleacea* [29], and *M. polymorpha* [32]. Here, we extend this list by evaluating telomere length in several new bryophyte species (*C. purpureus*, *Sphagnum* isolates), as well as in additional genotypes of model mosses and liverworts (*P. patens*, *M. polymorpha*). We show that the mean telomere length in all analyzed bryophytes is relatively short, typically below 2.5 and often below 1.5 kb. Although telomere length appears to be a heritable trait [53,54], it has been shown to vary drastically between geographically and genetically distinct populations of the same species in many eukaryotic lineages [41,53,55,56]. We further investigated this feature of telomere biology and found that TL variation is also common in bryophytes. Specifically, all four species investigated herein were found to harbor genotype-specific telomere length. Different ecotypes of *P. patens*, *C. purpureus*, *S. fallax*, and *M. polymorpha* are thought to have adapted to life in distinct environments throughout the world, and current efforts are underway to generate their full genome sequences. Thus, the observed substantial intra-species variation in telomere length can serve as a strong foundation for their future use in association mapping and quantitative trait loci studies to discover causal genetic variants.

### 3.2. Restriction Enzyme Choice and Detection of Interstitial Telomeric Sequences

The classical terminal restriction fragment analysis assay involves digesting genomic DNA with restriction enzymes that typically recognize four base pair DNA sites, like TT|AA for TruI1, which is the enzyme of choice for many plant telomere investigations. Interestingly, for most bryophyte samples analyzed here, we discovered that using a combination of two or three restriction enzymes (TruI1 and RsaI, or HaeIII, MboI, and AluI) produced better TRF fragment separation. Although the *P. patens* genome, for example, has relatively low GC content (34.6%) [57,58], we hypothesize that the combination of TruI1 (TT|AA) and RsaI (GT|AC) treatments likely results in a more complete digestion of degenerate subtelomeric sequences adjacent to the perfect TTTAGGG repeats at the ends of chromosomes. We conclude that for the short telomeric tracts observed in most analyzed bryophytes, the use of double and triple digests in TRF assays is recommended.

Telomere length data generated by the TRF method can also be affected by the presence of interstitial telomeric repeats (ITR or ITS) that hybridize with the telomeric DNA probes. Genomic regions with ITS are composed of telomeric sequences located in the internal regions of chromosomes and are found in genomes of many vertebrates [59,60], insects [61,62], yeast [63], and plants [64]. The presence of ITS in a bryophyte genome was first noted in the Gd ecotype of *P. patens* [30]. Here, we also detected the presence of ITS sequences in the Reute genome, suggesting that interstitially located telomeric repeats are relatively common in *P. patens* ecotypes. Interestingly, we also noticed the presence of a very strong ITS-like band in *S. fallax* TRF gel; however, pretreatment of *S. fallax* DNA with BAL31 nuclease largely abolished this signal, which may suggest its terminal location on the chromosome. Given that bands of similar size were also detected in TRF gels for two other *Sphagnum* isolates, the possibility of ITS presence in the *Sphagnum* genomes requires future investigation.

### 3.3. Telomere Length Variations in Dioecious Bryophytes

In humans at birth, females have on average longer telomeres than males, possibly contributing to the well-established differences in the average life expectancy between the sexes [65]. Indeed, in many animals, males and females often age at different rates [66], which initially led to a hypothesis that sex differences in telomere length could play a role in longevity variation in animals overall. However, no consistent sex differences in telomere length could be established between males and females in a very large panel of mammalian, bird, fish, and reptile species, suggesting that humans may be relatively unique with regard to this feature of chromosome biology [67]. In plants, separate sexes are characteristic of only 4 % of angiosperms, but in bryophytes, this number is remarkably high, over 50 % [24]. We measured TL in male and female strains of the two dioecious bryophytes, *M. polymorpha* and *C. purpureus*. In the four analyzed Ceratodon isolates, all lines showed substantial variation in TL, but no correlation with the sex of the plant strain could be established. Similarly, TL differences in the two tested male and female accessions of *M. polymorpha* were also identified, though more accessions need to be analyzed to support or reject the hypothesis of TL correlation with the plant sexes. Nevertheless, harnessing the unique and powerful constellation of the rapidly developing genomic tools and resources for model bryophytes will allow researchers to investigate genotype- and sex-specific telomere length regulation in the near future.

## 4. Materials and Methods

### 4.1. Plant Material

Axenic protonema of moss *Physcomitrium patens* Hedw., ecotype Gransden (Gd, Gransden Wood, Cambridge, UK), ecotype Villersexel-3 (Vx, Villersexel, Haute Saône, France), ecotype Reute (Re, Freiburg im Breisgau, Germany) [39], and ecotype Kaskaskia (Ka, Mississippi River Kaskaskia Island, IL, USA) were obtained from Prof. Stefan Rensing (Philipps-Universität Marburg). *Ceratodon purpureus* cultures GG1 (female isolate, Gross Gerungs region), B150 (female, AK, USA), R40 (male, NY, USA), and B190 (male, AK, USA) were obtained from Dr. Stuart McDaniel, University of Florida. Liverwort *Marchantia polymorpha* subsp. ruderalis cultivars Takaragaike-1 (male, Tak-1) and Takaragaike-2 (female, Tak-2) were obtained from Prof. Takayuki Kohchi (Kyoto University, Japan). Peat moss *Sphagnum fallax* H. Kliggr. isolate MW (MN, USA) was obtained from Dr. David Weston and Dr. Megan Patel, Oak Ridge National Laboratory. *Sphagnum girgensohnii* Russ. tissue was collected in the forest area near Ekaterinburg, Russia. *Sphagnum* sp. tissue was collected in a forest area in the Republic of Mari El, Russia. *Sphagnum* species identification was performed by comparative morphological and anatomical bryology methods with optical equipment, as described in the identification guides [68,69].

### 4.2. Plant Cultivation

*P. patens* and *C. purpureus* plants were propagated as axenic protonema and gametophore cultures on Petri dishes with BCD medium: 1 mM MgSO_4_, 1.84 mM KH_2_PO_4_ pH 6.5, 10 mM KNO_3_, 0.045 mM FeSO_4_, 1 mM CaCl_2_, and the trace elements of 9.93 mM H_3_BO_3_, 2.2 mM CuSO_4_ × 5H_2_O, 1.96 mM MnCl_2_ × 4H_2_O, 0.231 mM CoCl_2_ × 6H_2_O, 0.191 mM ZnSO_4_ × 7H_2_O, 0.169 mM KI, and 0.103 mM Na_2_MoO_4_ × 2H_2_O, supplemented with 5.5 mM ammonium tartrate and 0.7% agar [70]. Plants were passaged every 2 weeks on Petri plates with BCD covered by cellophane discs by protonema homogenization using an Ultra-Turrax T10 dispenser (IKA, Staufen im Breisgau, Germany). *S. fallax* MV gametophores were grown on plates with BCD medium and passaged monthly. *M. polymorpha* thalli or gemmae were propagated on Petri plates with Gamborg’s B5 medium containing 25 mM KNO_3_, 1 mM CaCl_2_ × 2H_2_O, 1 mM MgSO_4_ × 7H_2_O, 1 mM (NH_4_)_2_SO_4_, 1 mM NaH_2_PO_4_ × H_2_O and trace elements (0.003 g/L H_3_BO_3_, 0.01 g/L MnSO_4_ × H_2_O, 0.002 g/L ZnSO_4_ × 7H_2_O, 0.043 g/L Ferric-EDTA, 0.25 × 10^−3^ g/L Na_2_MoO_4_ × 2H_2_O, 0.025 × 10^−3^ g/L CuSO_4_ × 5H_2_O, 0.025 × 10^−3^ g/L CoCl_2_ × 6H_2_O, 0.75 × 10^−3^% KI), 2 mM MES, supplemented with 1% agar, pH 5.5 [71]. Plants were grown in the growth chamber (Klimatostat KS-200 SPU, Smolensk, Russia) at 16 h/8 h day/night light regime, temperature 22 °C/20 °C, 65% humidity, 880 lux light intensity.

### 4.3. DNA Extraction

Genomic DNA was extracted from 7- to 42-day-old protonema of *P. patens* and *C. purpureus*, the thalli of 21–28-day-old *M. polymorpha*, and from 28-day-old gametophores of *Sphagnum* isolates. Plant tissues were collected and grounded in mortars with pestles in liquid N_2_, and DNA extracted by the optimized CTAB buffer method [72]. Concentration and quality of DNA samples were analyzed using a DeNovix DS-11 Spectrophotometer (DeNovix, Wilmington, DE, USA) followed by an agarose gel confirmation.

### 4.4. Telomere Length Analysis

Telomere length was measured by terminal restriction fragments (TRF) analysis as described before [73,74] with minor modifications. Genomic DNA was digested with *Tru1l*; or combinations of step-wise digestion with TruI1 and RsaI (New England Biolabs, Ipswich, MA, USA); or HaeIII, MboI, and AluI enzymes, which are commonly used to analyze plant telomeric DNA [75]. The digested DNA samples were separated by gel electrophoresis in a 2% agarose gel at 55V for 18 h in 1X TAE buffer and transferred to a Hybond-N+ nylon membrane (GE Healthcare, Chicago, IL, USA). ^32^P-labeled or digoxigenin (DIG)-labeled (TTTAGGG)_4_ probes were used for telomeric DNA sequence detection. Radioactive signals were scanned with a Pharos FX Plus Molecular Imager (Bio-Rad, Hercules, CA, USA), and nonradioactive signals were scanned with a ChemiDoc XRS+ system (Bio-Rad). Images were visualized with Quantity One v.4.6.5 or Image Lab™ v.6.1 software (Bio-Rad, Hercules, CA, USA), and mean telomere length values (mean TRF) were calculated using the WALTER program [76]. BAL31 nuclease (New England Biolabs, Ipswich, MA, USA) digestions at 0, 30, 60, and 120 min intervals were performed as described before [30].

### 4.5. Statistical Analysis

Statistical analysis was carried out with GraphPad Prism v.8 software (San Diego, CA, USA). Mean TRF distribution was used for box-and-whiskers plots (Tukey’s plots). A *p* value < 0.05 (two-tailed Student’s *t*-test) was considered statistically significant.

## Data Availability

The data that support the findings of this study are available in the Supplementary Materials of this article.

## Supplementary Materials

The following supporting information can be downloaded at: https://www.mdpi.com/article/10.3390/plants13030387/s1, Figure S1. Telomere length variation in *C. purpureus* ecotypes. (A) TRF Southern blot for DNA from *C. purpureus* lines B150 (female), B190 (male), GG1 (female) and R40 (male) digested with TruI1. Molecular weight DNA markers (in kb) are shown. (B) Telomere length (mean TRF) distributions in ≥3 biological replicates of each genotype are shown in boxplots. Significant *p*-values are shown; ns - no significant differences; Figure S2. Analysis of sensitivity of *S. fallax* telomeric DNA to BAL31 nuclease. TRF Southern blot (A) and agarose gel (B) for BAL31-digested *S. fallax* MW DNA. Lanes show *Tru1I* digestion of *S. fallax* genomic DNA without prior BAL31 treatment (0 min), and after various incubation periods with BAL31 exonuclease (30, 60, 120 min). Molecular weight markers are shown in kb. WALTER software quantifications of signal intensity from telomeric DNA treated with BAL31 for 0 min (C), 30 min (D), 60 min (E), and 120 min (F) are shown. Red lines show signal intensity for 2.1 kb band; Figure S3. Telomere length dynamics in *C. purpureus* GG1 and R40 ecotypes. TRF Southern blots for DNA from 14-, 28- and 42-day protonema cultures of GG1 (A) and R40 (B) ecotypes digested with *Tru1I*. Telomere length (mean TRF) distributions in 3 biological replicates of GG1 (C) and R40 (D) cultures are shown in boxplots. No significant changes in telomere length are detected; Table S1. Telomere length distribution in *P. patens* ecotypes, in kb; Table S2. Telomere length distribution in *P. patens* Gd and Re ecotypes (in kb) treated with different enzyme combinations; Table S3. Telomere lengths distribution in *C. purpureus* isolates, in kb; Table S4. Telomere length distribution (in kb) in *C. purpureus* ecotypes treated with different enzyme combinations; Table S5. Telomere length distribution in *M. polymorpha* ecotypes, in kb; Table S6. Telomere length distribution in *Sphagnum* isolates, in kb.
