# Supplementary material for: Telomere Length Variation in Model Bryophytes
Source: Plants (Basel). 2024 Jan 28;13(3):387. doi: 10.3390/plants13030387 (PMC10856949; doi:10.3390/plants13030387)
Supplement: Supplementary file 1 [file plants-13-00387-s001.zip › plants-2778499-supplementary.pdf]

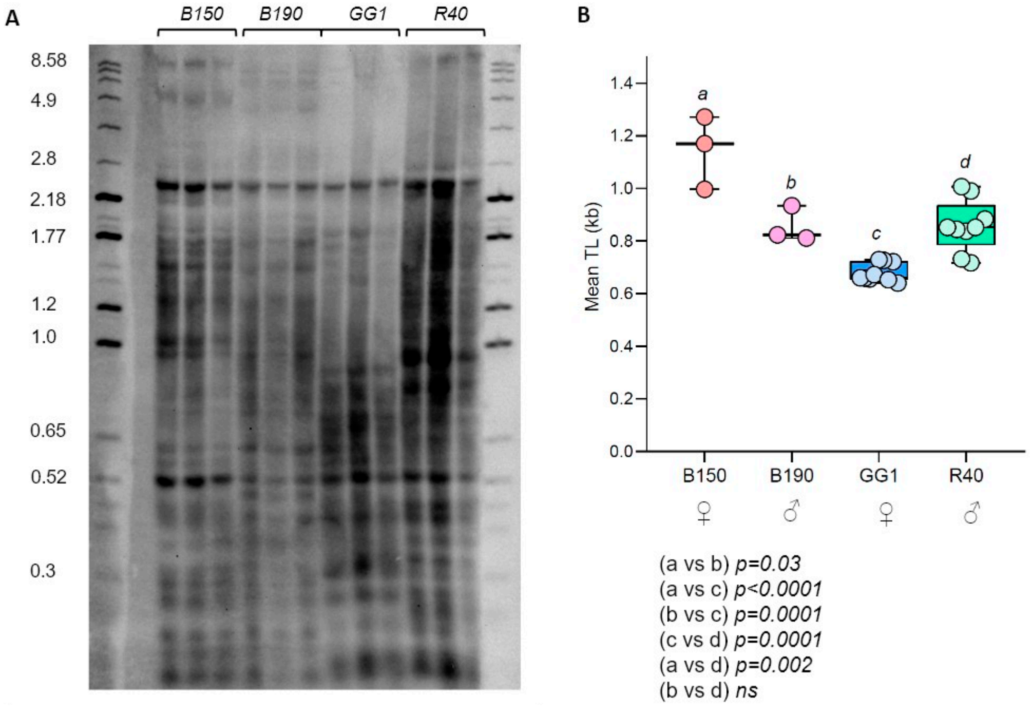

Supplemental Figure S1. Telomere length variation in *C. purpureus* ecotypes. (**A**) TRF Southern blot for DNA from *C. purpureus* lines B150 (female), B190 (male), GG1 (female) and R40 (male) digested with *TruI1*. Molecular weight DNA markers (in kb) are shown. **(B)** Telomere length (mean TRF) distributions in ≥3 biological replicates of each genotype are shown in boxplots. Significant *p*-values are shown; ns - no significant differences.

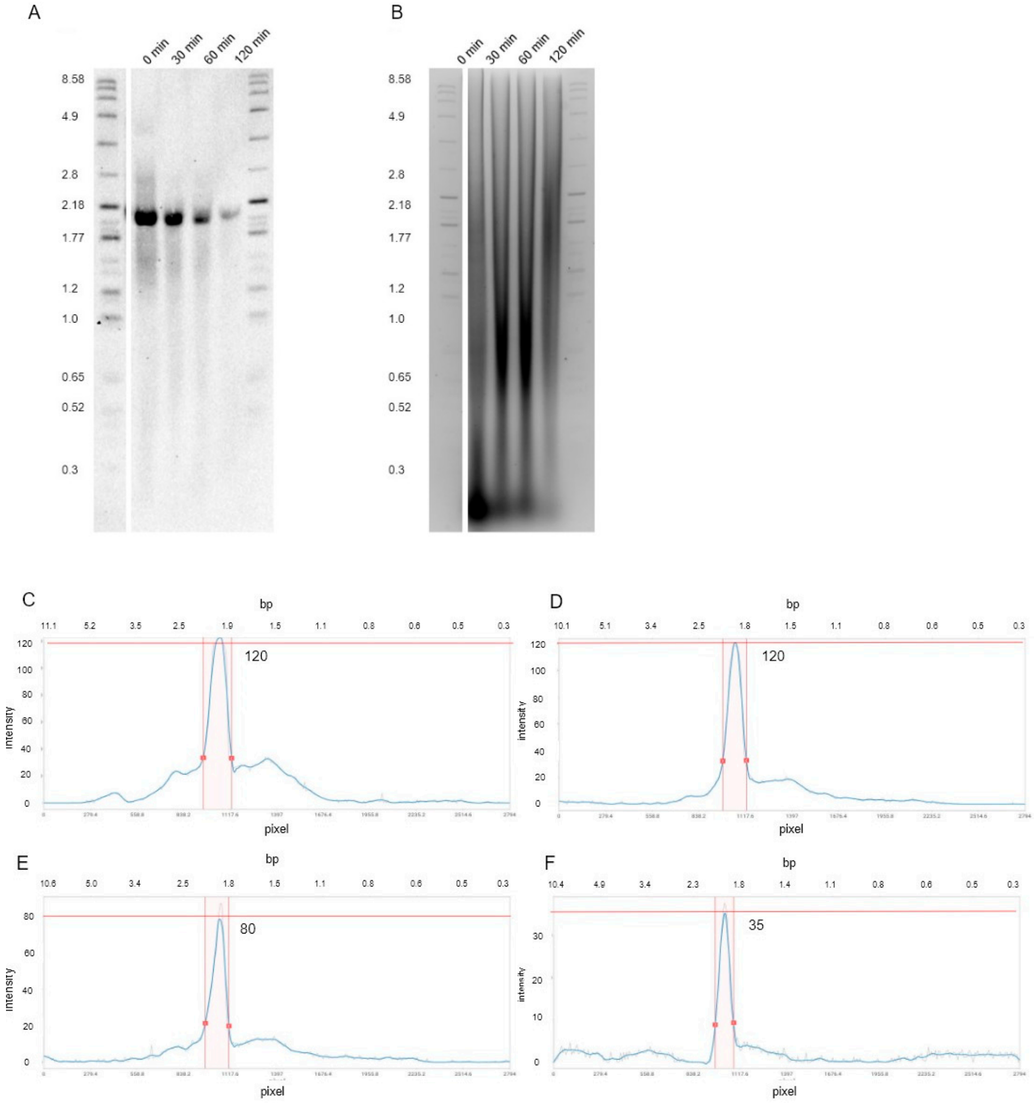

Supplemental Figure S2. Analysis of sensitivity of *S. fallax* telomeric DNA to *BAL31* nuclease. TRF Southern blot **(A)** and agarose gel (**B**) for *BAL31*-digested *S. fallax* MW DNA. Lanes show *Tru1I* digestion of *S. fallax* genomic DNA without prior *Bal31* treatment (0 min), and after various incubation periods with *Bal31* exonuclease (30, 60, 120 min). Molecular weight markers are shown in kb. WALTER software quantifications of signal intensity from telomeric DNA treated with *BAL31* for 0 min (**C**), 30 min (**D**), 60 min (**E**), and 120 min (**F**) are shown. Red lines show signal intensity for 2.1 kb band.

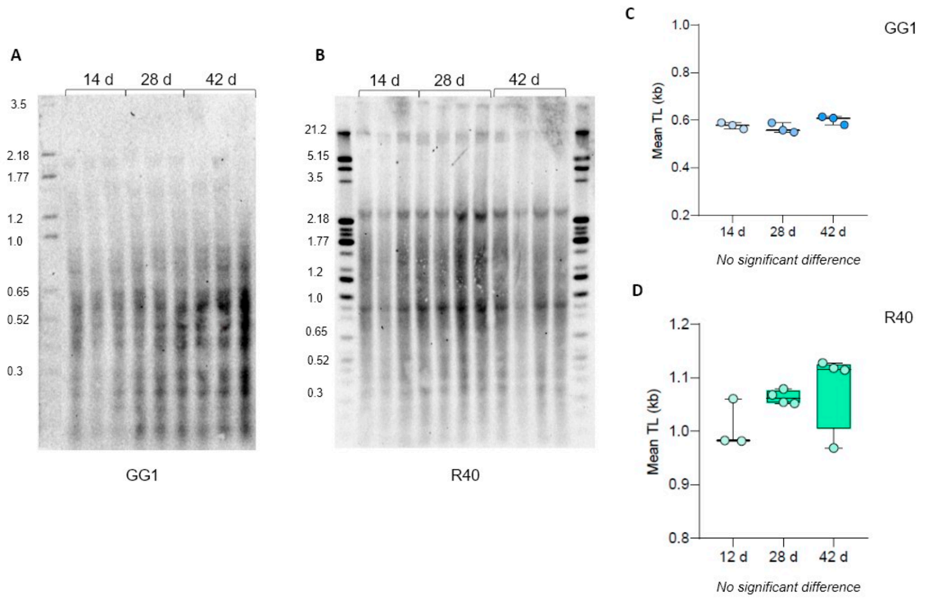

Supplemental Figure S3. Telomere length dynamics in *C. purpureus* GG1 and R40 ecotypes. TRF Southern blots for DNA from 14-, 28- and 42-day protonema cultures of GG1 (**A**) and R40 (**B**) ecotypes digested with *Tru1I*. Telomere length (mean TRF) distributions in 3 biological replicates of GG1 (**C**) and R40 (**D**) cultures are shown in boxplots. No significant changes in telomere length are detected.

Supplemental Table S1. Telomere length distribution in *P. patens* ecotypes, in kb.

Telomere length	Gd	Re	Ka	Vx
Mean	1.21±0.06	1.29±0.04	1.08±0.13	1,71±0,19
Min	0.67±0.03	0.88±0.12	0.70±0.07	0,95±0,18
1st Quartile	0.88±0.04	1.08±0.09	0.85±0.10	1,32±0,15
Median	1.14±0.04	1.27±0.04	1.03±0.14	1,67±0,18
3rd Quartile	1.62±0.13	1.53±0.13	1.34±0.19	2,14±0,34
Max	3.16±0.41	2.25±0.26	2.41±0.69	3,93±1,67

Supplemental Table S2. Telomere length distribution in *P. patens* Gd and Re ecotypes (in kb) treated with different enzyme combinations.

Telomere length	Gd		Re	
	TruI1 and *RsaI*	*HaeIII, MboI, AluI*	*TruI1* and *RsaI*	*HaeIII, MboI, AluI*
Mean	1.15±0.004	1.17±0.012	1.35±0.07	1.46±0.05
Min	0.79±0.02	0.88±0.001	1.04±0.11	1.14±0.03
1st Quartile	1.01±0.01	1.00±0.001	1.22±0.08	1.31±0.03
Median	1.15±0.004	1.15±0.006	1.34±0.06	1.44±0.04
3rd Quartile	1.30±0.008	1.35±0.03	1.48±0.07	1.63±0.07
Max	1.65±0.04	1.84±0.17	1.90±0.09	2.12±0.14

Supplemental Table S3. Telomere lengths distribution in *C. purpureus* isolates, in kb.

Telomere length	B150	B190	GG1	R40
Mean	1.15±0.14	0.86±0.07	0.68±0.04	0.85±0.10
Min	0.57±0.01	0.47±0.08	0.43±0.06	0.46±0.11
1st Quartile	0.90±0.14	0.65±0.07	0.58±0.04	0.63±0.09
Median	1.13±0.16	0.81±0.07	0.66±0.03	0.81±0.08
3rd Quartile	1.41±0.11	1.11±0.06	0.80±0.03	1.12±0.15
Max	2.20±0.04	2.38±0.18	1.41±0.42	1.86±0.46

Supplemental Table S4. Telomere length distribution (in kb) in *C. purpureus* ecotypes treated with different enzyme combinations.

Telomere length	GG1		R40	
	TruI1, RsaI	*HaeIII, MboI, AluI*	*TruI1, RsaI*	*HaeIII, MboI, AluI*
Mean	0.61±0.02	1.53±0.11	0.90±0.11	1.42±0.03
Min	0.48±0.01	1.14±0.03	0.52±0.06	1.11±0.11
1st Quartile	0.53±0.01	1.33±0.03	0.67±0.14	1.29±0.04
Median	0.60±0.02	1.47±0.07	0.89±0.15	1.39±0.03
3rd Quartile	0.70±0.04	1.79±0.23	1.14±0.05	1.59±0.04
Max	0.98±0.05	3.21±1.02	1.61±0.11	2.36±0.06

Supplemental Table S5. Telomere length distribution in *M. polymorpha* ecotypes, in kb.

Telomere length	Tak-1	Tak-2
Mean	2.15±0.25	2.45±0.25
Min	1.54±0.17	1.55±0.20
1st Quartile	1.83±0.21	1.87±0.20
Median	2.11±0.27	2.36±0.28
3rd Quartile	2.52±0.29	3.12±0.32
Max	3.41±0.40	4.64±0.21

Supplemental Table S6. Telomere length distribution in *Sphagnum* isolates, in kb.

Telomere length	*S. fallax*	*Sphagnum sp.*	*S. girgensohnii*
Mean	1.86±0.08	1.35±0.11	1.56±0.21
Min	1.12±0.21	1.06±0.01	0.91±0.16
1st Quartile	1.61±0.11	1.12±0.07	1.25±0.16
Median	1.91±0.10	1.33±0.10	1.54±0.23
3rd Quartile	2.06±0.06	1.60±0.16	1.88±0.25
Max	3.39±1.15	2.34±0.11	3.56±0.86
